# An irregular narrow complex tachycardia: atrial fibrillation or something else?

**DOI:** 10.1007/s12471-018-1217-y

**Published:** 2018-12-14

**Authors:** M. J. Mulder, C. P. Allaart, H. A. Hauer, M. J. B. Kemme

**Affiliations:** 1 Department of Cardiology, Amsterdam UMC, Amsterdam Cardiovascular Sciences, Vrije Universiteit Amsterdam, Amsterdam, The Netherlands; 2 Cardiology Centres of the Netherlands, Amsterdam, The Netherlands

## Answer

**Fig. 1:**
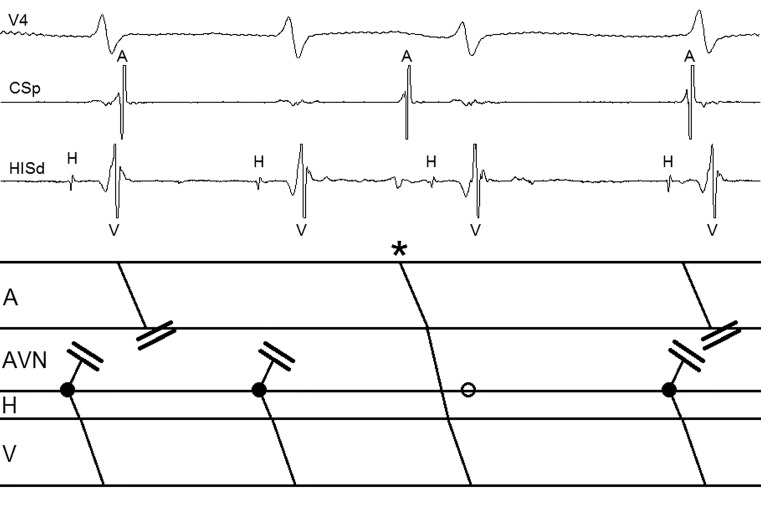
Tachycardia during electrophysiology study with corresponding ladder diagram. *Solid dots *represent ectopic foci from the atrioventricular junction. Note the lack of retrograde His-atrial conduction. The second sinus beat (*asterisk*) occurs prior to the anticipated junctional ectopic beat (*open circle*) and advances His bundle and ventricular activation without terminating or resetting the tachycardia. This response confirms the diagnosis of focal junctional tachycardia (*A* atrium, *AVN* atrioventricular node, *CSp* proximal coronary sinus, *H* His bundle, *HISd* distal His bundle, *V* ventricle)

The intracardiac recording during tachycardia (Fig. 1) shows a regular atrial rhythm, excluding atrial fibrillation. His bundle activation precedes ventricular activation and there is atrioventricular dissociation. The main differential diagnosis comprises atrioventricular node reentrant tachycardia (AVNRT), which may occur without atrial activation, and focal junctional tachycardia (FJT). Distinction is possible by analysing the response to a sinus beat which occurs before junctional depolarisation when the His bundle is non-refractory. Normal conduction of the sinus beat excludes AVNRT with bidirectional AH block. The sinus beat is conducted down the fast AV nodal pathway, leading to advancement of His bundle and ventricular activation without terminating or resetting the tachycardia. In common type (slow-fast) AVNRT, the subsequent refractoriness of the fast pathway would terminate the tachycardia. In contrast, termination is not expected during FJT, because of its focal nature [1]. Therefore, this tachycardia was diagnosed as FJT.

FJT is a rare arrhythmia in adults and is characterised by a rapid heart rate, narrow QRS complexes and atrioventricular dissociation. Frequently, an irregular rhythm is present which could lead to misdiagnosis of atrial fibrillation. The response to antiarrhythmic drugs is usually poor. Ablation is considered an effective alternative [2]. In this patient, after diagnosis of FJT, radiofrequency energy was delivered in the lower two-thirds of the Koch’s triangle, during which the tachycardia converted to sinus rhythm. Atrioventricular conduction was preserved. She remained free of symptoms during 3 months of follow-up.
